# A Mighty Claw: Pinching Force of the Coconut Crab, the Largest Terrestrial Crustacean

**DOI:** 10.1371/journal.pone.0166108

**Published:** 2016-11-23

**Authors:** Shin-ichiro Oka, Taketeru Tomita, Kei Miyamoto

**Affiliations:** Okinawa Churashima Foundation, 888 Ishikawa, Motobu, Okinawa 905–0206, Japan; Griffith University, AUSTRALIA

## Abstract

Crustaceans can exert a greater force using their claws than many animals can with other appendages. Furthermore, in decapods, the chela is a notable organ with multifunctional roles. The coconut crab, *Birgus latro*, is the largest terrestrial crustacean and has a remarkable ability to lift weights up to approximately 30 kg. However, the pinching force of this crab’s chelae has not been previously investigated. In the present study, we measured the pinching force of the chelae in 29 wild coconut crabs (33–2,120 g in body weight). The maximum force ranged from 29.4 to 1,765.2 N, and showed a strong positive correlation with body mass. Based on the correlation between pinching force and body weight, the force potentially exerted by the largest crab (4 kg weight) reported in a previous study would be 3300 N, which greatly exceeds the pinching force of other crustaceans as well as the bite force of most terrestrial predators. The mighty claw is a terrestrial adaptation that is not only a weapon, which can be used to prevent predator attack and inhibit competitors, but is also a tool to hunt other terrestrial organisms with rigid exteriors, aiding in these organisms to be omnivores.

## Introduction

Most decapod crustaceans possess well-developed chelae, which are typically used to capture, manipulate, and process prey (e.g., crush), as well as for defense and aggressive intraspecific interactions and displays [1]. Pinching forces of decapod chelae vary greatly and are largely determined by the claw size [1]. Notably, the chelae of decapods (e.g. *Cancer* spp.) can exert a greater pinching force relative to their body mass than other animals [2].

The coconut crab, *Birgus latro*, the largest terrestrial crustacean, has unique characteristics related to their phylogenetic position. The coconut crab shares a common ancestor with terrestrial hermit crabs, *Coenobita* spp., and has lost dependence on shells for the protection of the pleon in adult stages, and instead develops a calcified body [3]. Independence from shells removes restrictions on body size [4], and has possibly led to the development of new functions associated with their large claws.

The claw of the coconut crab can exert a tremendous amount of force. During our field study [5–7], obtaining data for analysis was challenging, as the large claws of this crab pinched us on multiple occasions. *B*. *latro* possesses a remarkable strength with a reported lifting force of up to 28 kg [8]. However, no previous study has reported the actual pinching force of this species, although a morphological study on crustaceans suggested that, among decapod crustaceans, the chelae of the coconut crab could generate the strongest pinching force [9]. In the present study, we aimed to (i) determine the pinching force of the wild coconut crab from measurement *in situ* and (ii) clarify how these animals acquired their mighty claw, using morphometric and ecological data.

## Materials and Methods

### Field protocol

**Fig 1:**
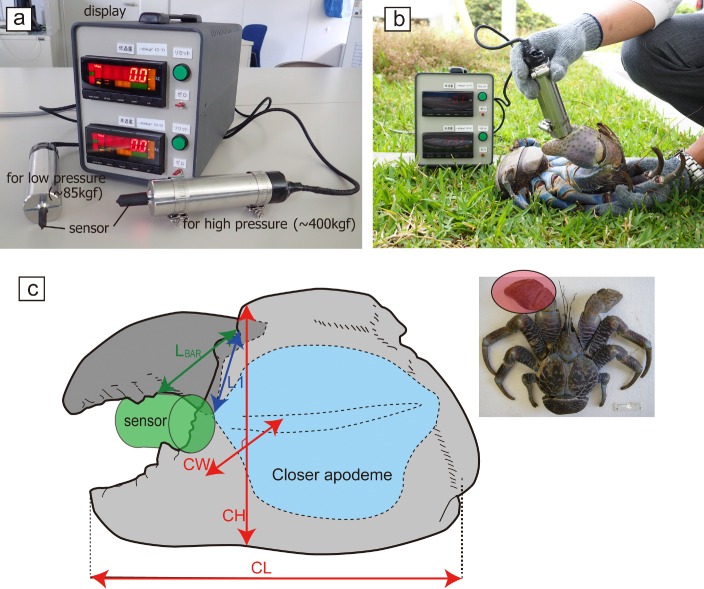
Measurement of the pinching force and claw morphology in the coconut crabs. (a) The force was measured with the SK-MBF-01F device (SkyScience Co. Tokyo, Japan) and related sensors and (b) demonstration of the method by which pinching force was measured. (c) Claw measurements of the coconut crab used in this study. The placement of the sensor used for pressure measurement is highlighted in green. The measurements used for claw length (CL), claw height (CH), and claw width (CW) are also indicated. L1: in-lever length from the fulcrum to the apodemes insertion; L_BAE_: out-lever length from the fulcrum to the tubercle (the contact point with the device sensor).

Pinching force measurements were performed on 29 coconut crabs collected from the northern part of Okinawa Island (Ocean Expo Park, 26°41′N, 127°52′E), located in the southern region of Japan. The thoracic length (ThL) and body weight (BW) ranged from 16.2–64.5 mm and 33.0–2120 g, respectively. The force exerted by the left chelae was measured for all crabs. Claw length (CL), claw height (CH), and claw width (CW) were measured to the nearest 0.1 mm using digital calipers for the left claws of 23 crabs (Fig 1C, S1 Table). The maximum pinching force at the largest tubercle on the fixed finger was measured using a bite force-measuring device (SKYSCIENCE, SK-MBF-01F; Fig 1A and 1B). This device can measure the force (kilogram-force, kgf) exerted by the crab while pinching a stainless steel stick-shaped sensor. Two sensors are available: 5 or 10 mm in diameter for low (approximately 85 kgf) and high (approximately 400 kgf) pressure measurements, respectively. Load cells are embedded in the sensor handle, which display an accurate measurement of the maximum force (kgf, nearest 0.1 kg) applied to main device. The pinching force measurements obtained in kgf were converted to newtons (N) by multiplying the kgf value by 9.807. All crabs were immediately released at the capture points after measurement. The field research was conducted with the permission of the Okinawa Commemorative National Government Park Office, which manages the park.

### Estimation of the closer muscle stress

**Fig 2:**
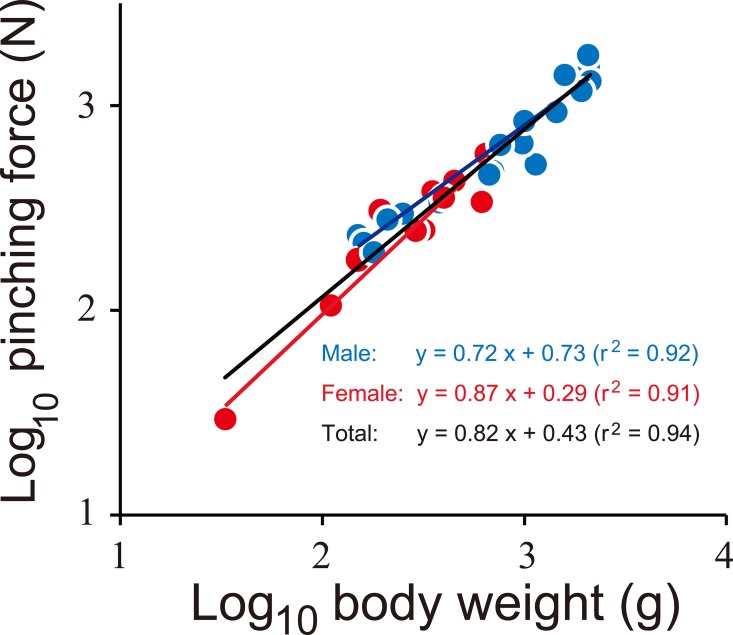
The relationship between body weight and pinching force of the coconut crabs. Blue and red indicate male and female, respectively. Black line is a regression line using the combined data of both sexes.

The closer muscle fiber sarcomere length was measured in the larger left claw of a voucher coconut crab specimen (OCF-Cr00051 deposited in the Okinawa Churashima Foundation, 51.3 mm ThL, male, preserved 70% ethanol solution after 10% formalin fixation). The muscle tissues (dorsal-ventral, mid-way along the manus) were embedded in paraffin, sectioned to 7 μm, and stained with phosphotungstic acid hematoxylin (PTAH) for histological observation. Single sarcomere lengths from a fiber on the slide were measured using a digital microscope (OLYMPUS, BX53). Muscle stress in this specimen was calculated using the following formula: *S* = *F/A*sin2*θ*, which was obtained in a previous study [10], where *F* is the force applied to the base of the dactyl, estimated from the relationship between the actual pinching force and body weight (Fig 2), and the mechanical advantage (L_ber_/L_1_, Fig 1C). The value of A (the area of one side of the closer apodeme) and *θ* (the mean angle of the fiber attached the closer apodeme) of the claw specimen were directly measured.

## Results and Discussion

**Fig 3:**
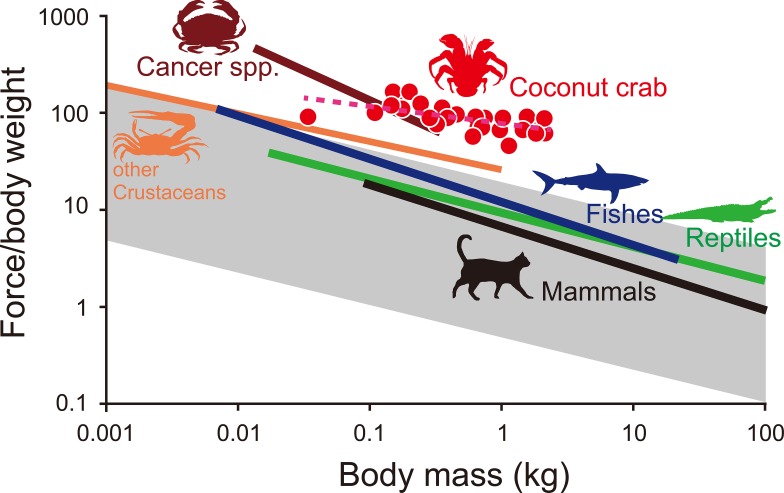
Regression analysis of the maximum force per unit body weight vs. body mass across several animal groups, including coconut crabs. The shaded gray area represents the range of the maximum force exerted by various animal activities (running, jumping, pushing, pulling, swimming, flight, nipping, and biting) [11]. Colored lines were calculated based on the relationship between the closing forces of crustacean chelae, vertebrate jaws and body masses determined previously [1, 2].

The pinching force exerted by coconut crabs was extremely strong. Maximum pinching force ranged from 29.4 to 1765.2 N. The scaling factor in the allometric equation for pinching force and BW was 0.82 (Fig 2). This value was significantly greater than the predicted isometric scaling of pinching force (proportional to muscle cross-sectional area) against BW (a = 0.67 [11]). According to a previous study, the reported maximum BW of the coconut crab is 4 kg [12]. Applying our allometric scaling equation, the pinching force of the coconut crab of 4 kg BW was estimated to be 3300 N. This force greatly exceeds that in all other crustacean species that have been reported [1, 2], as well as the bite force for the majority of modern terrestrial predators, other than alligators [13–14]. The maximum force exerted by major muscle groups (in terms of force / body weight) usually ranges between 10 body mass^-1/3^ and 50 body mass^-1/3^ [11] (Fig 3). Among the force associated with the closure of crustacean chelae and vertebrate jaws [14] few exceed the upper range of these values, while the pinching forces of *Cancer* spp. [2] and the coconut crab do exceed the upper limit. The relative pinching force of the coconut crab was greater than the force of any animal above 0.14 kg BW, although coconut crabs less than 0.14 kg BW were inferior to *Cancer* spp. (Fig 3).

**Fig 4:**
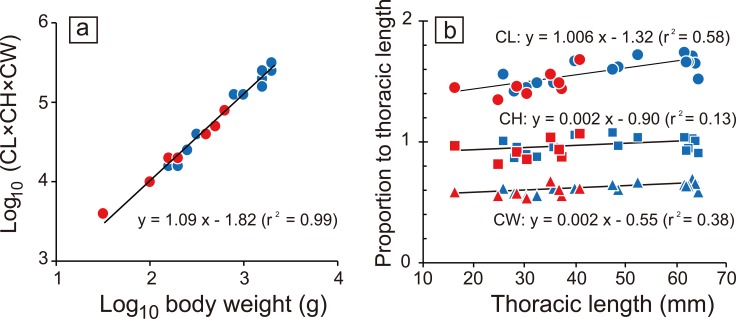
The relationship between (a) body weight and the index of claw-mass, multiplied by claw length (CL), claw height (CH), and claw width, and (b) the thoracic length and each claw size measurement (circle: CL, square: CH, triangle: CW). Blue and red indicate male and female, respectively. Black lines are regression lines based on the combined data of both sexes.

A morphological study of coconut crabs in Christmas Island, Indian Ocean, identified sexual dimorphism in chelae size and heterochely [15]. This study shows that the pinching forces of males and females might be expected to differ. However, contrary to the expectation, the regression line of pinching force and BW did not differ between the two sexes in the present study (ANCOVA, p = 0.58; Fig 2). In addition, sexual dimorphism of chela morphology was not found in the relationship of body size to claw measurements (claw-mass index, CL, CH and CW; Fig 4B; ANCOVA, p > 0.19). Thus, sexual dimorphism with respect to chelae morphology and pinching forces in coconut crabs was not found in the Okinawan population.

The strong claws of the coconut crab are likely associated with the loss of shelter it underwent through the course of its evolution. It is generally accepted that coconut crabs are derived from a hermit crab ancestor, which would have used a hard gastropod shell shelter; this evolutionary split likely occured during the Pliocene, 2.6–5.3 million years ago [16]. Like their close relatives in the genus *Coenobita*, the early juvenile stages of the coconut crab carry a shell for protection; however, at ca. 10 mm ThL, the thorax and pleon harden, and the crabs are no longer reliant on the shell for protection [12]. The loss of a shelter possibly led to an increase in body size and calcification of the body in order to avoid predation [3]. In addition, coconut crabs perform agonistic displays to maintain their solitary lifestyle [17], and often fight aggressively with each other and other animals for food and resources [12, 18]. The mighty claws of these crabs are useful weapons to deter predators and competitors. They can be used to enable the crabs to consume a wide variety of foods, including fruit, fallen tree pith, and various types of carrion [12]. Their mighty claws also allow them to be active predators by facilitating effective hunting and feeding on other terrestrial organisms with hard exteriors, thereby aiding in the maintenance of their large body size [19]. In particular, the ability of these crabs to open coconuts [12] demonstrates the impressive force of their claws.

The extreme pinching force exerted by crab claws may be based on geometrical changes and/or the sarcomere length of muscle fibers [1, 2]. However, the massive force of coconut crab chelae cannot be explained based solely on these two factors. Geometrical changes of the pinching force indicate that the larger the body size, the greater the pinching force (Fig 2). The claw-mass index (CL x CH x CW) was strongly correlated with BW (Fig 4A). We found that the scaling exponent (inclination) of 1.09 in the relationship between BW and the claw-mass index was not significantly different from the expected value of 1.0 (ANCOVA, p = 0.29), which would indicate isometric similarity. Additionally, the proportional length of each measurement (/ThL) remained almost unchanged throughout their ontogeny (Fig 4B). Thus, the proportional size and geometry of the claw of the crabs measured in this study have remained almost unchanged during ontogeny. The sarcomere length (mean ± SE) was 8.0 ± 0.18 μm (n = 70, S2 Table). The constants for the determination of muscle stress were estimated as: F = 949.9 N, A = 11.38 cm^2^, and *θ* ± SE = 48.24 ± 0.76° (n = 70). The maximum muscle stress was calculated as 829.4 kN/m^2^. Previous research demonstrated that the sarcomere length of crustaceans is greater than that of other animals, and the length is directly proportional to the maximum muscle stress [2]. Applying this correlation, the maximum muscle stress of the coconut crab requires a sarcomere length of 14 μm. However, the measured sarcomere length in the chelae of coconut crab averaged only 8 μm. Although the mystery of the massive force remained unsolved, further biochemical and/or physiological approaches (e.g. determination of ATPase activity in the muscle; density measurement of the muscle filaments; structure and formation of the muscle, as well as apodeme and carapace strength) might explain the strength of the pinching force in the coconut crab.

In summary, coconut crabs have the ability to exert the greatest force among almost all terrestrial animals. This animal is well adapted to terrestrial life in terms of sensory, respiratory, excretory, and osmoregulatory functions [4]. The powerful claw provides some advantages to their terrestrial life style. They can monopolize rigid terrestrial foods such as coconut, which are unavailable to other animals, and they can drive off predators and other competitors. Thus, the mighty claw is likely to be an adaptation to a terrestrial lifestyle and had been produced over the course of coconut crab evolution.

## Supporting Information

S1 Table Pinching force of the crabs including in this study, for the pinching force measurements. (PDF)

S2 Table Sarcomere length measurements. (PDF)
