# Supplementary material for: A Mighty Claw: Pinching Force of the Coconut Crab, the Largest Terrestrial Crustacean
Source: PLoS One. 2016 Nov 23;11(11):e0166108. doi: 10.1371/journal.pone.0166108 (PMC5120803; doi:10.1371/journal.pone.0166108)
Supplement: S1 Table — (PDF) [file pone.0166108.s001.pdf]

S1 Table. Pinching force of the crabs including in this study, for the pinching force measurements.

ThL (mm)	BW (g)	Sex	CL(mm)	CH(mm)	CW(mm)	Pinching force(kgf)
16.2	33	F	23.7	15.9	9.8	3.0
24.8	110	F	33.7	20.7	14.2	10.8
28.0	150	M	40.0	24.6	16.5	23.8
28.5	150	F	41.9	26.4	16.8	18.0
28.5	160	M	42.1	27.6	16.8	21.8
25.8	180	M	40.4	26.4	16.3	19.6
30.5	194	F	42.9	26.6	16.8	31.2
32.5	250	M	48.9	29.0	18.5	30.3
34.0	290	F	−	−	−	24.9
35.7	320	F	−	−	−	25.2
35.8	350	M	53.8	34.6	22.4	38.8
37.4	380	F	54.2	33.4	21.4	34.4
36.9	400	F	55.4	34.9	22.9	36.2
35.2	450	F	55.2	37.0	24.3	43.8
42.1	614	F	−	−	−	34.6
41.0	642	F	69.2	44.1	26.0	59.3
40.0	670	M	67.2	42.8	25.3	47.0
45.1	700	M	−	−	−	48.8
48.6	760	M	79.3	47.5	29.9	65.7
51.8	980	M	−	−	−	66.9
47.5	1000	M	76.4	51.9	31.5	85.6
49.8	1140	M	−	−	−	52.7
52.5	1440	M	90.6	55.1	35.6	95.0
62.1	1580	M	103.5	58.4	40.3	144.0
64.5	1770	M	98.5	59.1	38.4	134.5
62.5	1920	M	104.4	60.2	40.7	120.3
61.7	2070	M	107.7	64.9	40.8	180.0
63.3	2090	M	109.1	65.9	45.2	161.0
63.9	2120	M	106.0	65.1	43.0	134.5
