# Supplementary material for: A Mighty Claw: Pinching Force of the Coconut Crab, the Largest Terrestrial Crustacean
Source: PLoS One. 2016 Nov 23;11(11):e0166108. doi: 10.1371/journal.pone.0166108 (PMC5120803; doi:10.1371/journal.pone.0166108)
Supplement: S2 Table — (PDF) [file pone.0166108.s002.pdf]

S2 Table. Sarcomere length measurements.

sarcomere length (μm)			
1	8.06	36	6.41
2	8.37	37	6.41
3	7.43	38	6.55
4	7.29	39	10.00
5	7.22	40	8.46
6	7.26	41	9.84
7	6.71	42	9.97
8	6.66	43	9.71
9	9.47	44	10.08
10	9.14	45	10.53
11	7.68	46	11.44
12	7.76	47	9.19
13	7.96	48	9.76
14	7.56	49	7.17
15	6.75	50	8.21
16	7.19	51	9.21
17	9.39	52	8.74
18	7.80	53	11.00
19	8.19	54	8.19
20	7.39	55	7.40
21	7.07	56	8.06
22	6.32	57	6.23
23	6.83	58	7.53
24	5.56	59	9.29
25	6.01	60	7.03
26	5.68	61	8.52
27	5.68	62	9.16
28	7.48	63	8.62
29	6.87	64	9.81
30	7.49	65	8.53
31	5.96	66	9.52
32	5.47	67	9.97
33	5.37	68	9.50
34	6.60	69	10.24
35	7.43	70	10.13
